# Alterations in intestinal microbiota diversity, composition, and function in patients with sarcopenia

**DOI:** 10.1038/s41598-021-84031-0

**Published:** 2021-02-25

**Authors:** Lin Kang, Pengtao Li, Danyang Wang, Taihao Wang, Dong Hao, Xuan Qu

**Affiliations:** 1 Department of Geriatrics, Peking Union Medical College Hospital, No. 1 Shuaifuyuan, Dongcheng District, Beijing, 100730 China; 2 Allwegene Technology Inc., Beijing, 102209 China; 3 Department of Geriatrics, Urumqi Friendship Hospital, Urumqi, China; 4 Department of Geriatrics Centre, Hainan General Hospital, Haikou, China; 5 Department of Geriatrics, Liaocheng People’s Hospital, Liaocheng, Shandong Province China

**Keywords:** Microbiome, Musculoskeletal abnormalities, Next-generation sequencing, RNA sequencing

## Abstract

16S rRNA sequencing of human fecal samples has been tremendously successful in identifying microbiome changes associated with both aging and disease. A number of studies have described microbial alterations corresponding to physical frailty and nursing home residence among aging individuals. A gut-muscle axis through which the microbiome influences skeletal muscle growth/function has been hypothesized. However, the microbiome has yet to be examined in sarcopenia. Here, we collected fecal samples of 60 healthy controls (CON) and 27 sarcopenic (Case)/possibly sarcopenic (preCase) individuals and analyzed the intestinal microbiota using 16S rRNA sequencing. We observed an overall reduction in microbial diversity in Case and preCase samples. The genera *Lachnospira*, *Fusicantenibacter*, *Roseburia*, *Eubacterium*, and *Lachnoclostridium*—known butyrate producers—were significantly less abundant in Case and preCase subjects while *Lactobacillus* was more abundant. Functional pathways underrepresented in Case subjects included numerous transporters and phenylalanine, tyrosine, and tryptophan biosynthesis suggesting that protein processing and nutrient transport may be impaired. In contrast, lipopolysaccharide biosynthesis was overrepresented in Case and PreCase subjects suggesting that sarcopenia is associated with a pro-inflammatory metagenome. These analyses demonstrate structural and functional alterations in the intestinal microbiota that may contribute to loss of skeletal muscle mass and function in sarcopenia.

## Introduction

The human gut microbiome consists of a highly diverse, complex, and evolving community of microorganisms that colonize the length of the alimentary tract. Natural fluctuations in composition of the gut microbiome are most pronounced at the extremes of age, namely infancy and old age^1^. A number of studies have demonstrated robust differences in gut microbiota composition in older individuals when compared with younger adults^2,3^. Moreover, the gut microbiome of older adults demonstrates significant inter- and intra-individual variability, although there is a large degree of temporal stability in this population^4^. The dynamic nature of the microbiome is hypothesized to underly the physiologic and pathologic changes associated with aging. For example, dysregulation in older persons’ immunomodulatory abilities (referred to as immunosenescence or inflammaging) and resultant increase in pro-inflammatory cytokines including tumor necrosis factor (TNF)-α, interleukin (IL)-6, and IL-8, have been correlated with alterations in gut microbial composition^5,6^.

Frailty represents a syndrome or phenotype characterized by age-related decline in reserve and physiologic function across multiple organ-systems thus limiting individuals’ ability to tolerate minor stressors. An unfortunate, but inevitable, part of aging, frailty places individuals at increased risk for adverse health outcomes^7,8^. In skeletal muscle, frailty is manifested as sarcopenia, or the age-related decline in muscle mass and function. This loss of muscle function makes individuals of advanced age vulnerable to a number of devastating events including falls, fractures, disability, and mortality^9^. Sarcopenia and, more generally, physical frailty develop as a result of multiple pathophysiologic mechanisms including inadequate nutrition and physical activity, inflammation, immunosenescence, anabolic resistance, and oxidative stress^10–13^. These processes, particularly those related to inflammation and the immune system, are greatly influenced by the gut microbiome^14,15^. Indeed, several reports have correlated altered microbial diversity with poor physical performance in older patients^16–19^. One particular analysis determined that the microbial profile of older community dwellers meeting criteria for physical frailty was similar to that of nursing home patients, implicating microbial dysbiosis in age-related performance decline^17^. Further implicating the role of the gut microbiome in age-related decline, Buigues et al. demonstrated a beneficial effect of a prebiotic supplement containing inulin and fructooligosaccharides on two-measures of frailty, exhaustion and handgrip strength (which is also a clinical parameter for sarcopenia)^20^.

Preclinical data has strengthened the association between gut dysbiosis and age-related physical frailty and performance decline and has led to the hypothesis that a gut-muscle axis exists through which the microbiome influences whole body lean mass, skeletal muscle mass, and physical functioning^21^. By this model, variation in the gut microbiota and gut metagenome alters key biological processes such as the inflammatory milieu (for example, by increased permeability of the gut epithelial barrier)^22^, nutrient bioavailability^23^, and lipid metabolism^24^, and can contribute to age-related muscle decline. Although human data is limited, a number of studies in animal models support this hypothesis. In their pioneering work, Backhed et al. demonstrated that germ-free mice colonized with fecal samples from their conventionally-raised counterparts display a significant loss in lean body mass – which includes skeletal muscle mass^25^. This is contrasted by a recent comparison of pathogen-free and germ-free mice which demonstrated skeletal muscle atrophy, reduced expression of insulin-like growth factor 1, and reduced expression of genes corresponding to skeletal muscle growth and mitochondrial function in the germ-free cohort^26^. Taken together, these data suggest that the microbial influence on skeletal muscle is likely taxa dependent. Specific bacterial species shown to support skeletal muscle growth and function include *Lactobacillus casei*, *Bifidobacterium longum*, *Eubacterium rectale*, *Clostridium coccoides,* and *Barnesiella intestinihominis*^27–29^. The proposed mechanism by which these species enhance skeletal muscle physiology is through the production of short chain fatty acids (SCFAs) such as acetate, propionate, and butyrate^21^. Siddarth et al. have also demonstrated that lean body mass and gastrocnemius muscle mass decline with aging in direct relation to circulating levels of vitamin B12 and lipid metabolism, implicating a role for these processes in age-related muscle decline^30^. In contrast, expansion of pro-inflammatory species, such as those within the large *Enterobacteriaceae* family of gram-negative bacteria, is associated with poor skeletal muscle integrity^31^. Proposed mechanisms underlying the effect of these bacteria on skeletal muscle include inflammation-mediated epithelial permeability, circulation of bacterial toxins (*i.e.*, lipopolysaccharide, LPS; indoxyl sulfate), and resultant insulin resistance and skeletal muscle atrophy^32^.

The development and availability of culture-independent approaches for evaluating microbial diversity, namely 16S ribosomal RNA (rRNA) sequencing, has fostered a surge in research on the human gut microbiota in recent years^33^. This research has provided an unprecedented understanding of the development, composition, and temporal stability of the gut microbiome while also identifying alterations associated with an array of pathologic processes^34,35^. While these approaches have been utilized to correlate microbial diversity with skeletal muscle mass and function in animal models and young adult humans, there are few published studies exploring microbiome alterations associated with physical frailty and muscular decline in the aged^16,29,36,37^. Moreover, to the best of our knowledge, no reports evaluating the gut microbiome in clinical sarcopenia exist. In this study, utilizing 16S rRNA analysis, we identified notable differences in microbial diversity between healthy older adults and those with sarcopenia. In addition to an overall reduction in microbial diversity in sarcopenia patients, our analysis revealed reduction in several butyrate-producing species providing novel insight into potential mechanisms by which microbial alteration may influence disease pathogenesis.

## Results

### Characteristics of study subjects

A total of 60 healthy controls (CON) and 27 sarcopenic (Case) or possibly sarcopenic (preCase) individuals were enrolled in this study. The criteria for defining sarcopenia (Case) and possible sarcopenia (preCase) are described in the Materials and Method section: briefly, Case individuals demonstrated impaired muscle function (*i.e.*, grip strength, five-time chair test) and reduced muscle mass (by bioelectrical impedance analysis, BIA), while preCase individuals demonstrated only impaired function. Detailed demographic and clinical characteristics, including BIA muscle mass and performance on muscle function tests, are summarized for each group in Table 1. There was a slight, but statistically significant (p = 0.001), trend towards advanced age in the preCase and Case groups when compared to controls. There was also a significant (p < 0.05) reduction in albumin and hemoglobin/hematocrit in the preCase and Case groups. Finally, consistent with reduction of muscle mass and function in sarcopenia, body mass index (BMI), appendicular skeletal mass index (ASMI), and grip strength, were significantly reduced in preCase and Case individuals while the time required to complete the five-time chair stand test was delayed.

**Table 1:** Clinical and demographic parameters of enrolled subjects. All values are reported as mean ± standard deviation unless otherwise noted.

	Sarcopenia (Case)	Possible sarcopenia (preCase)	Healthy control (CON)	P	F/χ^2^
Number of subjects	11	16	60		
Age in years	76.45 ± 8.58	74.00 ± 6.94	68.38 ± 5.79	0.000	F = 10.436
Number of females (%)	n = 7 (63.64%)	n = 11 (68.75%)	n = 33 (55.00%)	0.576	χ^2^ = 1.102
Albumin (g/L)	40.39 ± 3.98	40.46 ± 4.17	45.85 ± 2.50	0.000	F = 29.135
Creatinine (μmol/L)	73,115 ± 18.55	70.49 ± 14.22	66.75 ± 11.54	0.254	F = 1.393
Blood urea Nitrogen (mmol/L)	5.71 ± 2.29	5.81 ± 1.86	5.26 ± 1.08	0.316	F = 1.167
C-reactive protein (mg/L)	1.68 ± 1.74	2.13 ± 2.54	2.08 ± 3.09	0.923	F = 0.080
Hemoglobin (g/L)	122.91 ± 17.84	125.06 ± 18.98	141.00 ± 12.90	0.000	F = 12.081
HCT (%)	36.76 ± 4.50	36.93 ± 5.03	42.00 ± 3.0	0.000	F = 18.029
BMI (kg/m^2^)	20.67 ± 3.27	21.50 ± 3.39	23.66 ± 2.49	0.001	F = 7.846
Appendicular Skeletal Mass index (kg/m^2^)	6.97 ± 1.38	6.43 ± 1.22	7.84 ± 0.78	0.000	F = 15.401
Grip strength (kg)	22.49 ± 9.67	21.76 ± 6.35	29.57 ± 8.09	0.001	F = 8.196
Five-time chair stand test (s)	18.69 ± 14.09	15.40 ± 7.89	10.03 ± 1.13	0.000	F = 12.724

All values are reported as mean ± standard deviation unless otherwise noted. Significant values highlighted in yellow.

### Changes in overall microbial diversity and structure in sarcopenia

A total of 11,997,848 high-quality sequences were generated from 87 fresh human fecal samples with an average of 137,906 sequences per sample (Supplementary Table S1). High-quality sequences were clustered into 4,001 OTUs at 97% sequence identity. Next, a modified OTU table was obtained consisting of 3,856 OTUs (ranging from 35 to 1208 per sample) corresponding to 507 genera, 249 families, 145 orders, 93 classes, and 36 phyla. Adequate estimation of microbial diversity was ensured by measurement of the proportion of total bacterial species represented in samples from each group utilizing the Goods coverage analysis (Supplementary Fig. S1). Goods coverage values close to 1 (0.94–0.99, Case group; 0.98–0.99, preCase group; 0.96–0.99, CON group) suggested that the 16S rRNA sequencing results from each library represented the majority of bacterial species present within test samples.

**Figure 1:**
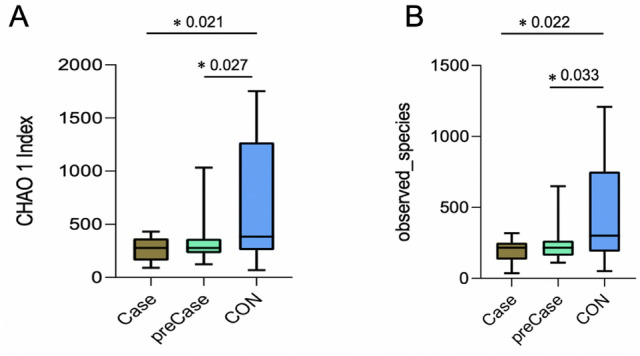
Alpha-diversity indices (**A**) Chao1 and (**B**) observed species are reduced in sarcopenic (Case) and possibly sarcopenic (preCase) samples compared to healthy control (CON) samples. Kruskal–Wallis test, *p < 0.05.

**Figure 2:**
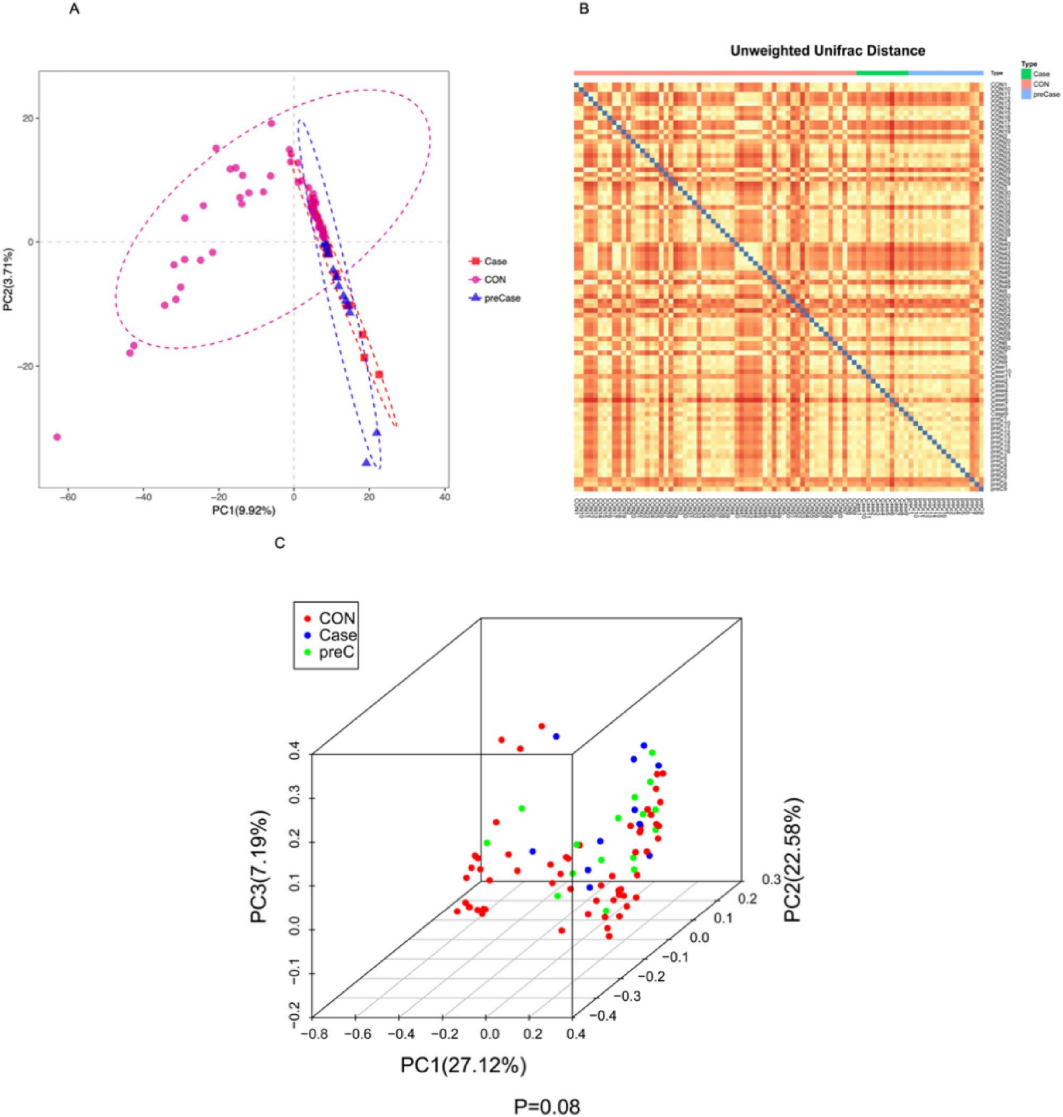
Beta-diversity indices identify significantly different microbial compositions between healthy controls (CON), sarcopenic (Case), and possibly sarcopenic (preCase) subjects. (**A**) Partial least squares discriminant analysis (PLS-DA) displays clustering of points (representing individual samples) by group and groups are elliptically defined. (**B**) A heat map generated from the unweighted-Unifrac distance matrix. (**C**) A three-axis principle components analysis (PCoA) of unweighted-Unifrac distances.

Within-sample species richness and evenness was evaluated, and differences were detected between healthy control (CON), sarcopenic (Case), and possibly sarcopenic (preCase) subjects. The alpha diversity indices of Chao1 and observed species diversity are displayed in Fig. 1. Both the Chao1 and observed species diversity indices were both significantly higher in the CON group compared to the Case and preCase groups (al-Wallis test, both p < 0.05; Fig. 1A,B). There was no significant difference between Case and preCase samples (p = 0.916, Chao1; p = 0.907, observed species). These results demonstrate significant reduction in microbial diversity in Case and preCase groups. Differences in microbial composition between Case, preCase, and CON samples were detected using beta-diversity analyses. Phylogenetic variation between the microbial communities of CON, Case, preCase groups was revealed by generation of a partial least squares discrimination analysis (PLS-DA; Fig. 2A)^38^. There was significant divergence between the CON and Case (*ANOSIM,* R = 0.370, p = 0.0001) and CON and preCase (*ANOSIM*, R = 0.272, p = 0.0001) groups. In contrast, the Case and preCase groups demonstrated substantial phylogenetic closeness (*ANOSIM*, R = 0.048, p = 0.187). Between-group differences were further evaluated by calculating UniFrac distances. Unweighted-Unifrac analysis found that the microbial composition of Case and PreCase subjects was close to significantly different from CON subjects (p = 0.08; Fig. 2B). A three-axis principle components analysis (PCoA) plot is presented in Fig. 2C. Taken together, reduced microbial diversity in sarcopenic and possibly sarcopenic individuals and significant differences for microbial composition between healthy individuals and sarcopenic/possibly sarcopenic individuals suggest that the pathogenesis of sarcopenia may be strongly influenced by intestinal microbial factors.

### Bacterial abundance and functionality in sarcopenia

**Figure 3:**
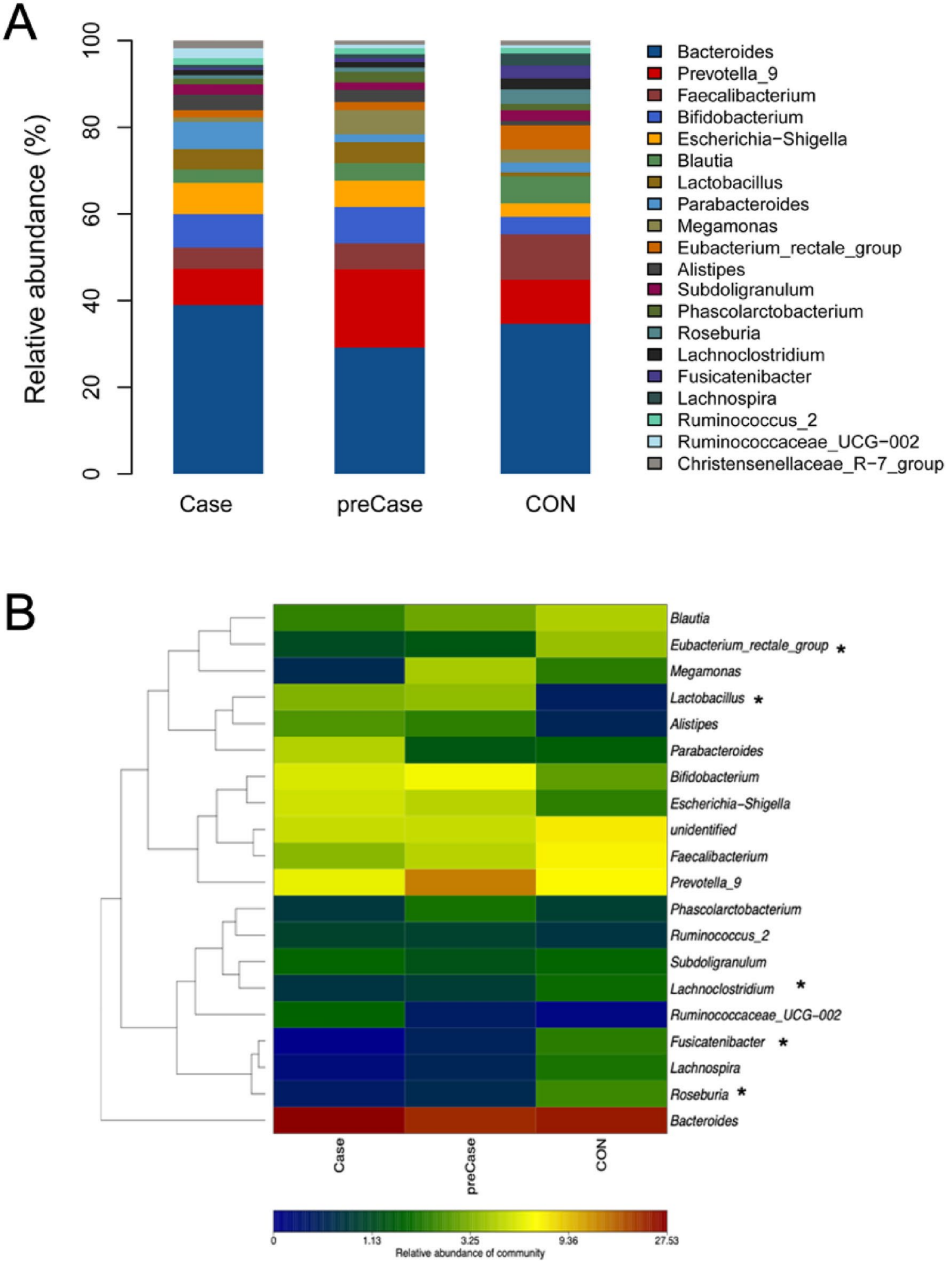
Taxonomic distribution of intestinal microbiota is altered in sarcopenic (Case) and possibly sarcopenic (preCase) subjects compared to healthy controls (CON). (**A**) Stacked columns for each of the groups show the mean of abundance for a given genus as a percentage of the total. (**B**) Heat map demonstrating the relative abundance of genera in each group. Wilcoxon test, * p < 0.05.

**Figure 4:**
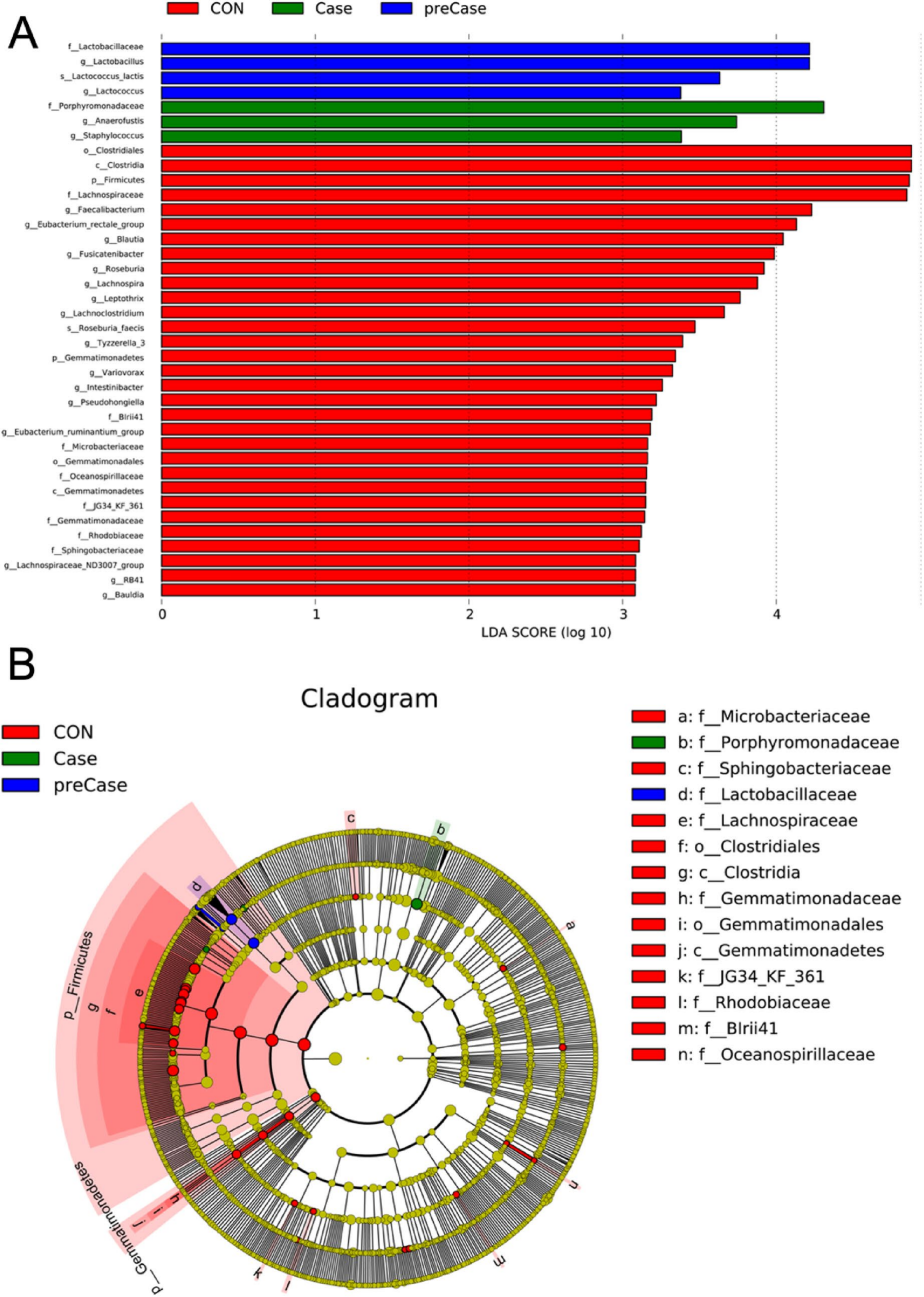
Taxonomic biomarkers of sarcopenia. (**A**) Linear discriminant analysis effect size (LEfSe) analysis comparing sarcopenic (Case) and possibly sarcopenic (preCase) patients with healthy controls (CON). (**B**) Cladogram displaying differentially abundant taxonomic clades with LDA score > 3.0 in Case, PreCase, and CON subjects.

Having identified significant differences in the microbial diversity and structure of sarcopenic (Case) and possibly sarcopenic (preCase) individuals, we then analyzed the abundance and taxonomic distribution of microbiota in these groups using Metastats. Indeed, we determined that the bacterial profiles of individuals in the Case and preCase groups was significantly different from that of the CON group. At the phylum level, the gut microbiota was dominated by *Firmicutes* (50.8%), *Bacteroidetes* (36.3%) *Proteobacteria* (6.4%) and *Actinobacteria* (5.1%) across the samples in our study. Notably, *Firmicutes* were significantly reduced in both the Case (40.4% vs 54.4%, p = 0.005) and preCase (44.5% vs. 54.4%, p = 0.005) groups compared to controls. There was no significant difference observed between the Case and preCase groups (40.4% vs 44.5%, p = 0.521). At the genus level, *Lachnospira*, *Fusicantenibacter*, *Roseburia*, *Eubacterium*, and *Lachnoclostridium* were significantly reduced, whereas *Lactobacillus* was significantly more abundant in the Case and preCase groups compared to the CON group (all p < 0.05; Fig. 3). These differentially abundant taxa were further analyzed as taxonomic biomarkers of sarcopenia using LEfSe (linear discriminative analysis, LDA = 3; Fig. 4). This analysis identified the bacterial families *Porphyromonadaceae* and *Lactobacillaceae* as taxonomic biomarkers of sarcopenia and possible sarcopenia, respectively.

### Clinical correlations of the microbiome with sarcopenia characteristics

**Figure 5:**
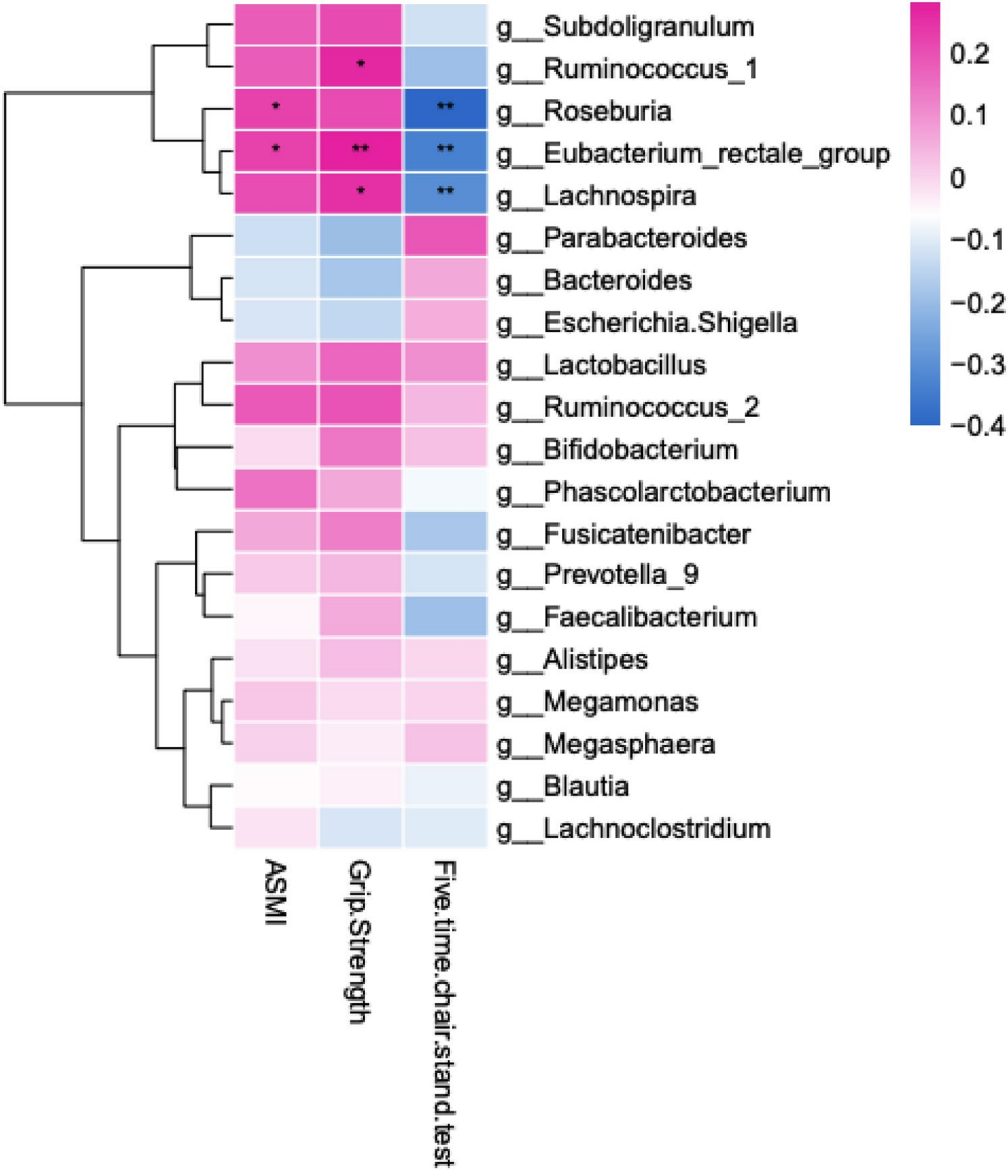
Heatmap showing correlations between microbiota genera and sarcopenic characteristics. Abundance (based on sequence counts) of microbiota genera correlate with muscle mass (ASMI) and functional muscle tests (grip strength, five-time chair stand test). The intensity of the color represents the correlation with the corresponding characteristics (negative correlation, blue; positive correlation, pink). ASMI, appendicular skeletal muscle index. Spearman test, *p < 0.05, **p < 0.01.

Correlation analysis was performed between gut microbiota genera abundances and clinical characteristics of sarcopenia, namely muscle mass (ASMI via BIA) and function (grip strength and five-time chair stand test). Slight, but significant (p < 0.05) positive correlation was identified between *Roseburia* and *Eubacterium* and ASMI, indicating that these genera are less abundant in individuals with smaller muscle mass. *Lachnospira* (p < 0.05), *Eubacterium* (p < 0.01), and *Ruminococcus* were significantly positively correlated with grip strength, while *Roseburia, Eubacterium,* and *Lachnospira* were negatively correlated (p < 0.01) with five-time chair stand test time. This indicates that the abundance of these genera decrease in proportion with declining muscle function, in addition to reduced muscle mass (Fig. 5).

### Predictive functional analysis

**Figure 6:**
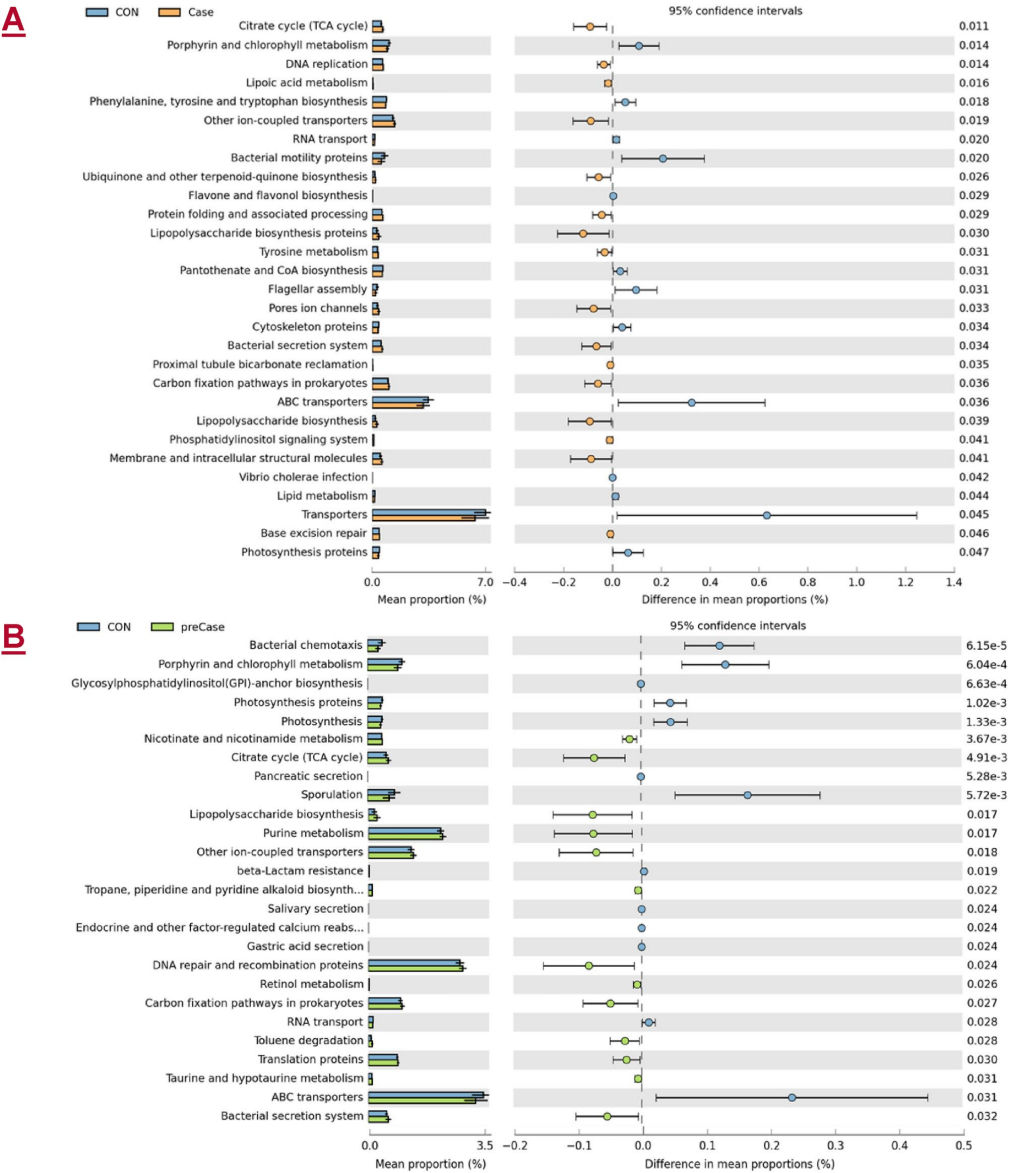
Selected pathways predicted by PICRUSt functional classification of the metagenome content of the microbiota in (**A**) sarcopenic (Case; yellow) and (**B**) possibly sarcopenic (preCase) versus healthy control (CON; blue) subjects. Welch’s t-test.

We next sought to understand how the taxonomic variations between the Case, preCase, and CON groups may contribute to or be functionally influenced by sarcopenia. We employed the PICRUSt program which utilizes 16S rRNA reads to predict differentially expressed functional and metabolic pathways (Fig. 6 & Supplementary Figs. S2, S3)^39^. A number of pathways were overrepresented in preCase and Case subjects including the citrate cycle, lipopolysaccharide biosynthesis, and protein folding and associated processing. Underrepresented pathways in Case subjects include cytoskeletal proteins, transporters, ABC transporters, and phenylalanine, tyrosine, and tryptophan biosynthesis. These results suggest that key metabolic pathways related to cellular energy production, protein processing, and nutrient transport are differentially regulated in the pathologic setting of sarcopenia. In addition, enrichment of LPS biosynthesis and LPS biosynthesis proteins suggests that sarcopenia is associated with a pro-inflammatory metagenome. Overall, our results indicate that factors associated with sarcopenia influence both microbiota composition and functional diversity.

## Discussion

The gut microbiota has emerged as a new and exciting frontier for understanding physiological changes associated with aging and pathophysiologic changes in disease states. While a number of independent studies have hypothesized a gut-muscle axis, thereby providing a potential link between the microbiome and age-related muscle decline, no studies have directly explored the microbiota composition in human patients with sarcopenia. In this study, we used high-throughput 16S rRNA sequencing of fecal samples from 27 individuals meeting diagnostic criteria for sarcopenia/possible sarcopenia and 60 healthy controls to demonstrate, for the first time, microbial taxonomic and predicted functional variations in this disease process. At baseline, the sarcopenic, possibly sarcopenic, and control groups were similar in gender distribution, renal function, and inflammatory status. While blood factors including albumin, hemoglobin, and hematocrit were lower in the preCase and Case groups, this is consistent with previous reports demonstrating reduced blood protein levels in relation to decline in muscle mass^40–42^. While the mechanism underlying the loss of albumin and hemoglobin in sarcopenic and possibly sarcopenic individuals is unclear, it may represent a more general impairment in protein metabolism (i.e., reduced stores, declining synthesis, and enhanced catabolism) that is related to muscle aging^43^. A potential discrepancy in the groups is the slight, but significant, trend towards older age in the preCase and Case groups. This is likely due to the elimination of individuals with significant comorbidities from the healthy control group, thus biasing this group towards a younger composition. Similarly, since sarcopenia is an age-related process, it is reasonable that the preCase and Case groups would be biased towards older age. While potentially confounding our results with general age-related microbial alterations, it was essential to eliminate individuals from the control group in whom comorbidities may more strongly influence their microbiome structure.

Overall taxonomic diversity, assessed by alpha-diversity indices, was found to be reduced in sarcopenic (Case) and possibly sarcopenic (preCase) individuals. While microbial diversity is known to be reduced with aging^44^, our comparison of the Case and preCase samples with healthy controls (CON) suggests that aging-related reduction in diversity is exaggerated in the setting of sarcopenia. A number of events, exposures, and mechanisms accompanying aging are thought to underly the reduction in microbial diversity, including a rise in antibiotic and other prescription drug use, changes in dietary pattern, hospitalization and communal (*i.e.*, nursing home) residency, chronic disease, and long-term, low-grade inflammation^5,45,46^. Interestingly, these conditions overlap with several risk factors for sarcopenia including chronic illness, malnutrition, sedentary lifestyle, polypharmacy, and nursing home residency^47–50^. More recently, in a cohort of female twins, Jackson et al. demonstrated robust associations between gut microbiota composition and frailty. Strikingly, microbial diversity demonstrated a strong inverse correlation with frailty index scores^19^. Further, in a randomized controlled trial, prebiotic supplementation was capable of reducing the burden of frailty suggesting that reduced microbial diversity may indeed be causative of physical decline^20^. Taken together with our results correlating reduced microbial diversity with sarcopenia, this suggests that the aging process may disrupt the microbiome and disturb gut-muscle axis homeostasis.

Our beta-diversity analyses identified an interesting reduction in the abundance of butyrate-producing genera in sarcopenic and possibly sarcopenic subjects These genera including *Lachnospira*, *Fusicantenibacter*, *Roseburia*, *Eubacterium*, and *Lachnoclostridium* all share a common ability to produce SCFAs, one of the most common of which is butyrate, a critical molecule through which microbiota influence host physiology^51–54^. Importantly, SCFAs are bacterial factors thought to attenuate inflammation and contribute to maintenance of skeletal muscle mass. Young germ-free mice fed a mixture of SCFAs (namely, acetate, propionate, and butyrate) displayed an elevated muscle mass:body weight ratio compared to those fed a control diet^26^. Walsh et al. further demonstrated that supplementation of butyrate in mice aged 16 months afforded substantial protection from hindlimb muscle atrophy via inhibition of histone deactylase^55^. SCFAs also appear to increase skeletal muscle function with several studies demonstrating improved exercise capacity or strength after SCFA supplementation^26,56,57^. Indeed, in our study these genera correlated slightly, but significantly, with muscle mass and functional parameters. These results suggest that a direct relationship exists between loss of these butyrate-producing bacteria and progression of muscle loss and impairment in sarcopenia. The decline in these bacteria is consistent with the negative association of age with abundance of SCFA-producing species in septuagenarians and super-centenarians previously reported^58^. Surpisingly, the butyrate-producer *Faecalibacterium prausnitzii*, which has been identified as less abundant in frail and older individuals in multiple previous studies^6,19,59^, was not found to be significantly reduced in our cohort. Moreover, subdominant genera and families including *Eggerthella, Akkermansia, Anaerotruncus, Synergistaceae, Bilophila,* and *Christensenellaceae* which have demonstrated increasing age-related trajectory in a previous analysis were not identified as enriched in our study^58^. While sarcopenia is an age-related disorder, and there are general microbiome-related processes that are altered in both aging and sarcopenia (i.e., butyrate production), these disparate results suggest that specific enriched or underrepresented taxa may be unique to each distinct process. One taxa that did demonstrate an increase in sarcopenic patients was the bacterial *Porphyromonadaceae* family, which emerged as a taxonomic biomarker of sarcopenia. Although less well characterized than the SCFA-producing bacteria, genera within this family (including *Parabacteroides* and *Porphyromonas*) have been associated with metabolic syndrome in both mouse models and humans^60,61^. In particular, the species *Porphyromonas gingivalis* relates to elevated glycemic levels in type two diabetics^62^. This suggests that *P. gingivalis* may impair insulin sensitivity, a mechanism postulated to underly sarcopenia^63^. Interestingly, the *Lactobacillus* genus, which is found in many probiotic formulations, was identified as a biomarker of the preCase group. One mechanism by which *Lactobacillus*-containing probiotics exert beneficial effects appears to be through ameliorating chronic, systemic inflammation. For example, *Lactobacillus* strains have been shown to reduce concentrations of the bacterial-derived endotoxin, lipopolysaccharide and related inflammatory factors^64,65^. The selective overrepresentation of this genus in preCase, but not Case subjects, suggests that reduction in inflammatory-modulating bacterial species may contribute to the progression of skeletal muscle decline. This is consistent with the known impact of chronic inflammation on the development of sarcopenia^10^.

PICRUSt analysis identified functional metabolic and physiologic pathways that are predicted to be over- or underrepresented in the Case, preCase, and CON samples. A number of key transport and amino acid synthesis pathways were underrepresented in Case samples compared to healthy controls. Of note, ATP-binding cassette (ABC) transporters, which are responsible for uptake of a number of substances including polar and hydrophobic amino acids^66,67^. The phenylalanine, tyrosine, and tryptophan biosynthesis pathways were also underrepresented in samples from sarcopenic individuals. Previous work has shown that essential amino acids – which include phenylalanine (and, by proxy, its derivative tyrosine) and tryptophan – are necessary for stimulation of muscle protein anabolism in older patients^68^. Because these amino acids are not produced naturally within the body, their ingestion and production by microbiota are thereby critical for maintenance of skeletal muscle. Therefore, reduction in their biosynthetic pathways may represent one mechanism contributing to sarcopenia pathogenesis. Conversely, a number of pathways were predicted to be overrepresented in Case samples, including the upregulation of LPS biosynthesis in the sarcopenic group. LPS is a gram-negative, bacteria-derived endotoxin that induces systemic inflammation. In rodents, LPS injection was shown to induce muscle catabolism through inflammatory signaling (*i.e.*, upregulation of circulating TNF-α and muscle activation of NF-κB) and reduction in the concentration of circulating and muscle IGF-1^69^. Interestingly, the pathways predicted in our analysis match closely to those identified in a recent preclinical analysis of the rat sarcopenia microbiome including pathogenic secretion systems (*i.e.,* LPS), ABC transporters, and dietary metabolism^30^. Taken together, these functional results suggest that dysbiosis accompanying sarcopenia induces major shifts in anabolic-catabolic balance and inflammation.

It is important to note the limitations of this study, particularly those relating to the generalizability of our findings. The number of Case and preCase (n = 27) subjects was considerably smaller than the number of CON (n = 60) subjects. As our definition of sarcopenia is reliant on the recently released Asian Working Group for Sarcopenia 2019 Consensus Report^70^, we have been limited in enrolling subjects according to this new criteria. Fortunately, we were able to obtain a large number of reads from all groups and identified statistically significant differences in microbial diversity, composition, and function. In addition, subjects enrolled in this study were geographically located in Beijing, China. It is well known that geographic location and cultural differences (*i.e.*, dietary patterns) contribute to varying microbiota structure and composition^71^. Even within similar geographic locations, the microbiome of individuals can vary based on their residence within the community versus skilled nursing facilities^4^. Caution must, therefore, be exercised in generalizing the results of our single institution trial to the larger global community. Overall, our data should be recognized as preliminary evidence that supports a role for the microbiota in sarcopenia. Future, more highly powered studies including subjects from multiple geographic regions are necessary. The reliance of our study on 16 s rRNA, and not metagenomic, data also limits our ability to identify enrichment of functional pathways based on presence of encoding genes. Rather, we used PICRUSt, which predicts functional pathways based on differentially represented bacterial species^39^. Future analyses employing a shotgun metagenomics approach or whole genome sequencing of bacteria would provide further insight into alterations in microbiome functional profiles occurring in sarcopenia and aging. Finally, the lack of independent cohort replication and/or functional preclinical validation make it unclear if consistent changes in the microbiota are causative or a result of the physiologic muscle decline that characterizes sarcopenia. Preclinical studies involving depletion and/or supplementation of taxa identified in this analysis are warranted to establish a causative effect. Additional clinical studies should assess the impact of pre- or probiotic supplementation on muscle mass and function in the older population.

In conclusion, this study provides a new, preliminary perspective on the influence of gut microbial variations on sarcopenia in a cohort of Chinese individuals of advanced age. The composition of the gut microbiota was significantly altered in older individuals that met diagnostic criteria for sarcopenia and possible sarcopenia when compared to healthy controls. Future studies are necessary to establish causal relationships and to determine if probiotic supplementation may be protective against sarcopenia, and more generally, physical frailty.

## Materials and methods

### Ethics statement

The protocol for this prospective pilot study to identify differences in the microbiome between sarcopenic, possibly sarcopenic, and healthy subjects of advanced age was approved by the Ethics Committee of the Peking Union Medical College Hospital, attached to the Chinese Academy of Medical Sciences (No. ZS-1586) on May 22, 2018. Approval was also obtained from the Institutional Review Board of the Institute of Basic Medical Sciences, Chinese Academy of Medical Sciences (Approval Number: 009–2014, 031–2017). The protocol is registered online at the Chinese Clinical Trial Registry (No. ChiCTR1800017115). All participants were made aware of the objectives, procedures, and potential risks of the study, and provided informed consent prior to enrollment. All study procedures were performed in accordance with the World Medical Association Declaration of Helsinki Ethical Principles for Medical Research Involving Human Subjects.

### Study participants

A prospective study was conducted from September 2018 to September 2019 during which 60 healthy controls and 27 patients with sarcopenia or possible sarcopenia (according to Asian Working Group for Sarcopenia 2019 Guidelines)^70^ were continuously recruited at the Peking Union Medical College Hospital in Beijing, China. Inclusion criteria of subjects were as follows: an age ≥ 60 years old, stable health, and ability to be autonomous. Possible sarcopenia was defined as: (1) grip strength < 28 kg (male) or < 18 kg (female); OR (2) five-time chair stand test ≥ 12 s. Sarcopenia was defined as muscle mass < 7.0 kg/m^2^ (male) or < 5.7 kg/m2 (female) by BIA, in addition to meeting criteria for possible sarcopenia above. Exclusion criteria of subjects were an inability to move and stand independently from a chair, neurological or musculoskeletal disease preventing performance of resistance exercise at home, chronic cardiopulmonary insufficiency preventing normal daily activities (either New York Heart Association Functional Classification III/IV or inability to tolerate 6-min walk test), renal insufficiency (creatinine clearance < 60 mL/min) or severe liver damage (transaminase levels elevated on more than two occasions) requiring limited protein intake (< 0.8 g/kg/day), or the presence of a malignant neoplastic disease. Exclusion criteria were based on factors known or likely to impact the intestinal microbiota or those that confound the diagnosis of sarcopenia^9,72^. Healthy controls were selected from the Peking Union Medical College Aging Cohort of Willing Donation to provide an approximately age-matched cohort. Healthy was defined as the absence of significant comorbidities including diabetes mellitus, gastrointestinal disease, hyperlipidemia, coronary artery disease, chronic obstructive pulmonary disease, and hepatorenal insufficiency. Baseline clinical and demographic values were compared via ANOVA and Chi-square for parametric and non-parametric data, respectively.

### Sample collection, DNA extraction, and 16S rRNA sequencing

Fecal samples (several grams) were collected into sterile 2 mL vials containing DET (dimethyl sulfoxide) buffer in accordance with the field sampling protocols of Allwegene Technology, Beijing, China. Samples were stored at − 80 °C until further analysis. Total bacterial genomic DNA was extracted from fecal samples using the PowerSoil DNA Isolation Kit (MoBio Laboratories, CA, USA) following manufacturer’s instructions. The integrity and size of the DNA were checked by gel electrophoresis on 0.8% agarose gels. The universal primers 338F (5′- ACTCCTACGGGAGGCAGCAG-3′) and 806R (5′- GGACTACHVGGGTWTCTAAT-3′) were used for polymerase chain reaction (PCR) amplification of the V3-V4 hypervariable regions of the bacterial 16S rRNA gene to comprehensively define the bacterial composition and abundance in healthy controls and possibly sarcopenic/sarcopenic patients. The PCR products were mixed in equidensity ratios and then the mixed products were purified with the AxyPrep Gel Extraction Kit (Axygen Biosciences, NJ, USA). Sequencing libraries were generated using the NEBNext Ultra DNA Library Prep Kit for Illumina (New England Biolabs, MA, USA) following manufacturer’s recommendations with index codes added. Library quality was assessed on the Qubit 2.0 Fluorometer (Thermo Fisher Scientific, MA, USA) and Agilent Bioanalyzer 2100 system (Agilent Technologies, CA, USA). Library sequencing was performed on the IlluminaMiSeq PE300 platform (Illumina, CA, USA) at Allwegene Technology, Beijing, China and 300 bp paired-end reads were generated. Sequencing of the 87 samples yielded a total of 11,997,848 high-quality reads. On average, each sample yielded 137,906 sequences, ranging from 36,088 to 649,884.

### Sequencing, bioinformatics, and statistical analyses

The analysis of sequence reads was conducted using Quantitative Insights Into Microbial Ecology (QIIME, v1.8.0, http://qiime.org/). Briefly, raw sequences with exact matches to the barcodes were assigned to respective samples and identified as valid sequences. Primer sequences and barcodes were removed and sequences underwent quality control. Paired-end reads were merged using Fast Length Adjustment of Short reads (FLASH; 1.2.0)^73^, sequences were denoised using USEARCH (v.8.0.1623)^74^, and chimera were checked with UCHIME^75^. The remaining high-quality sequences were clustered into operational taxonomic units (OTU) at 97% sequence identity using the UPARSE pipeline by VSEARCH (v.2.13.3)^38^. Clustering resulted in 4,001 OTUs, initially. Subsampling at a depth of 29,298 reads per sample to control for the differential sequencing effort left 3,856 OTUs (ranging from 35 to 1208). Sequences were aligned with MAFFT (v7.427) and taxonomy was assigned to the lowest possible taxonomic level using the Ribosomal Database Project (RDP) Classifier at a 70% bootstrap value threshold. The numbers of sequences were normalized for further analyses with the upper limit of rarefaction depths (− e 14,649) used as the cut-off value.

Alpha-diversity indices (observed species diversity and Chao1) were computed using QIIME. Kruskal–Wallis tests were used to identify significant between-group differences for alpha-diversity indexes between groups. Taxonomic abundances at the phylum and genus levels were analyzed using the Metastats function in Mothur^76^. To detect differentially abundant taxa between groups metagenomic biomarker discovery was performed using the linear discriminant analysis effect size (LEfSe) program (http://huttenhower.sph.harvard.edu/galaxy/root/index)^77^. Spearman correlation analysis was performed to identify species associated with sarcopenia clinical features (ASMI, grip-strength, and five-time chair stand test). The function of intestinal microbiota was predicted with the use of the phylogenetic investigation of communities by reconstruction of unobserved states program (PICRUSt, http://picrust.github.io/picrust/)^39^. Functional modules were compared between sarcopenic, possibly sarcopenic, and control subjects with STAMP v2.1.3 (http://kiwi.cs.dal.ca/ Software/STAMP) using Welch’s t-test. Statistical analyses were performed using the R software package.

## Supplementary Information

Supplementary Information.

## Data Availability

The datasets generated during and/or analysed during the current study are available in the National Institues of Health (NIH) Sequence Read Archive (SRA), https://www.ncbi.nlm.nih.gov/sra (Project Accession: PRJNA691136).
