# Supplementary material for: Alterations in intestinal microbiota diversity, composition, and function in patients with sarcopenia
Source: Sci Rep. 2021 Feb 25;11:4628. doi: 10.1038/s41598-021-84031-0 (PMC7907362; doi:10.1038/s41598-021-84031-0)
Supplement: Supplementary file 1 — Supplementary Information. [file 41598_2021_84031_MOESM1_ESM.docx]

**Alterations in Intestinal Microbiota Diversity, Composition, and Function in Patients with Sarcopenia**

Lin Kang, Pengtao Li, Danyang Wang, Taihao Wang, Dong Hao,Xuan Qu

**Supplementary Table S1.** Reads and Operational Taxonomic Units (OTUs) of 87 Fecal Samples

| **Sample ID** | **Raw Reads** | **Clean reads** | **Final Reads** | **Operational Taxonomic Units (OTUs)** |
| --- | --- | --- | --- | --- |
| Case1 | 133684 | 130898 | 29298 | 232 |
| Case2 | 318608 | 306846 | 29298 | 221 |
| Case3 | 111134 | 107304 | 29298 | 198 |
| Case4 | 402254 | 384114 | 29298 | 309 |
| Case5 | 87700 | 82700 | 29298 | 125 |
| Case6 | 232578 | 221866 | 29298 | 35 |
| Case7 | 388144 | 363490 | 29298 | 245 |
| Case8 | 113086 | 112058 | 29298 | 134 |
| Case9 | 247994 | 242258 | 29298 | 232 |
| Case10 | 90516 | 86038 | 29298 | 122 |
| Case11 | 45152 | 42826 | 29298 | 259 |
| preC1 | 704484 | 649884 | 29298 | 111 |
| preC2 | 223000 | 214100 | 29298 | 118 |
| preC3 | 447812 | 421924 | 29298 | 232 |
| preC4 | 284500 | 276388 | 29298 | 305 |
| preC5 | 111520 | 108608 | 29298 | 172 |
| preC6 | 262118 | 249608 | 29298 | 275 |
| preC7 | 45466 | 41652 | 29298 | 428 |
| preC8 | 85714 | 65152 | 29298 | 632 |
| preC9 | 84280 | 82198 | 29298 | 147 |
| preC10 | 215170 | 203928 | 29298 | 205 |
| preC11 | 454602 | 410136 | 29298 | 141 |
| preC12 | 295886 | 280418 | 29298 | 220 |
| preC13 | 231450 | 225586 | 29298 | 240 |
| preC14 | 79700 | 77116 | 29298 | 175 |
| preC15 | 118008 | 113396 | 29298 | 245 |
| preC16 | 214302 | 201884 | 29298 | 172 |
| CON1 | 49172 | 47236 | 29298 | 155 |
| CON2 | 42052 | 36088 | 29298 | 855 |
| CON3 | 49610 | 45584 | 29298 | 112 |
| CON4 | 256808 | 248030 | 29298 | 213 |
| CON5 | 128538 | 125376 | 29298 | 244 |
| CON6 | 110548 | 92184 | 29298 | 363 |
| CON7 | 92972 | 78976 | 29298 | 883 |
| CON8 | 106576 | 103922 | 29298 | 209 |
| CON9 | 46260 | 45196 | 29298 | 161 |
| CON10 | 119398 | 113324 | 29298 | 186 |
| CON11 | 152752 | 120234 | 29298 | 895 |
| CON12 | 105378 | 81380 | 29298 | 977 |
| CON13 | 74990 | 60662 | 29298 | 694 |
| CON14 | 52562 | 42310 | 29298 | 208 |
| CON15 | 148392 | 142186 | 29298 | 257 |
| CON16 | 169650 | 163710 | 29298 | 143 |
| CON17 | 100440 | 81766 | 29298 | 900 |
| CON18 | 74552 | 61670 | 29298 | 859 |
| CON19 | 40902 | 36672 | 29298 | 347 |
| CON20 | 378156 | 162792 | 29298 | 420 |
| CON21 | 249940 | 237854 | 29298 | 198 |
| CON22 | 87266 | 83636 | 29298 | 172 |
| CON23 | 100870 | 96536 | 29298 | 105 |
| CON24 | 188816 | 178690 | 29298 | 158 |
| CON25 | 43268 | 41810 | 29298 | 99 |
| CON26 | 119128 | 90240 | 29298 | 1068 |
| CON27 | 137604 | 126134 | 29298 | 221 |
| CON28 | 171130 | 132358 | 29298 | 924 |
| CON29 | 48308 | 48042 | 29298 | 54 |
| CON30 | 180482 | 173098 | 29298 | 183 |
| CON31 | 172576 | 164290 | 29298 | 321 |
| CON32 | 110570 | 104884 | 29298 | 214 |
| CON33 | 105340 | 82320 | 29298 | 1030 |
| CON34 | 292636 | 271946 | 29298 | 332 |
| CON35 | 69786 | 68118 | 29298 | 355 |
| CON36 | 137176 | 121820 | 29298 | 210 |
| CON37 | 105850 | 87816 | 29298 | 356 |
| CON38 | 47916 | 40122 | 29298 | 297 |
| CON39 | 267866 | 255348 | 29298 | 300 |
| CON40 | 119450 | 99810 | 29298 | 650 |
| CON41 | 51486 | 38180 | 29298 | 1208 |
| CON42 | 101098 | 85060 | 29298 | 653 |
| CON43 | 68704 | 60534 | 29298 | 660 |
| CON44 | 151242 | 124364 | 29298 | 865 |
| CON45 | 73856 | 49886 | 29298 | 768 |
| CON46 | 133408 | 129810 | 29298 | 227 |
| CON47 | 57438 | 55900 | 29298 | 150 |
| CON48 | 72828 | 59910 | 29298 | 1063 |
| CON49 | 200186 | 189538 | 29298 | 329 |
| CON50 | 215394 | 189302 | 29298 | 147 |
| CON51 | 59372 | 48480 | 29298 | 1001 |
| CON52 | 241906 | 208572 | 29298 | 503 |
| CON53 | 49674 | 47716 | 29298 | 61 |
| CON54 | 73152 | 56096 | 29298 | 863 |
| CON55 | 207594 | 197460 | 29298 | 274 |
| CON56 | 113138 | 98046 | 29298 | 281 |
| CON57 | 61496 | 55014 | 29298 | 182 |
| CON58 | 87568 | 84362 | 29298 | 163 |
| CON59 | 74860 | 62988 | 29298 | 579 |
| CON60 | 62922 | 60084 | 29298 | 211 |

**Supplementary Figure S1.** Goods coverage analysis measuring the proportion of total bacterial species represented in samples for each group. Values close to 1 (0.94-0.99, Case; 0.98-0.99, Pre-Case; 0.96-0.99, CON) confirm that the libraries generated from each group represent the majority of bacterial species present.


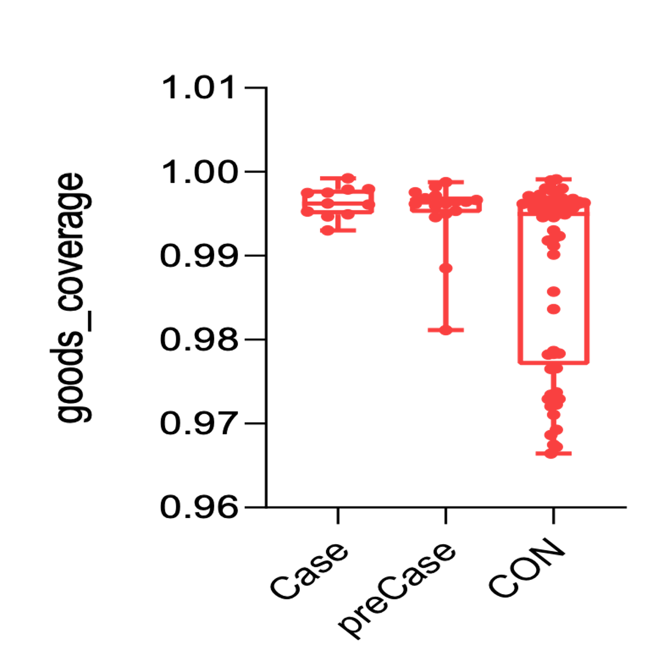


**Supplementary Figure S2.** All pathways predicted by PICRUSt in sarcopenic (Case; yellow) versus healthy control (CON; blue) subjects. Welch’s t-test.


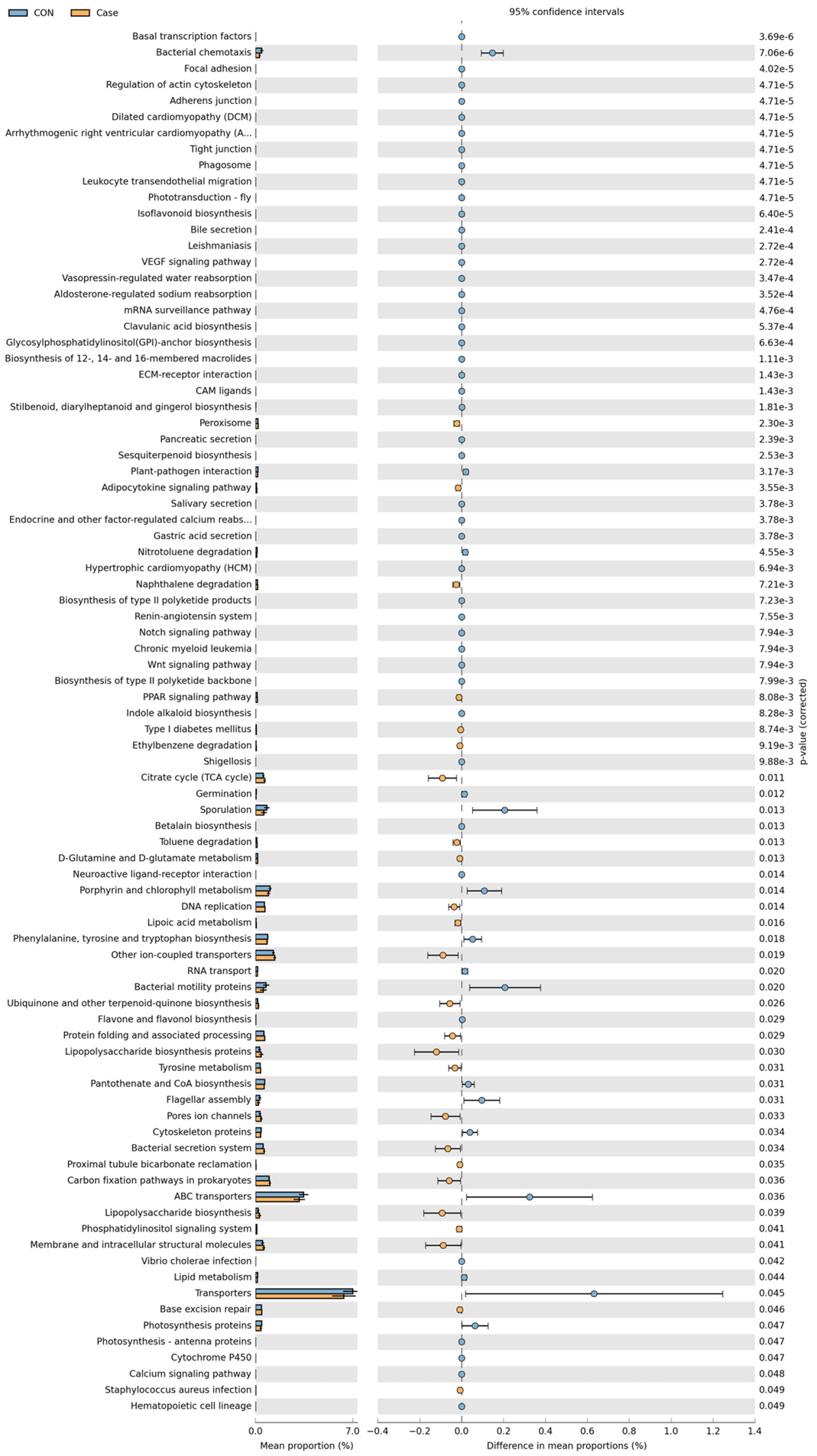


**Supplementary Figure S3.** All pathways predicted by PICRUSt in possibly sarcopenic (preCase) versus healthy control (CON; blue) subjects. Welch’s t-test.

**
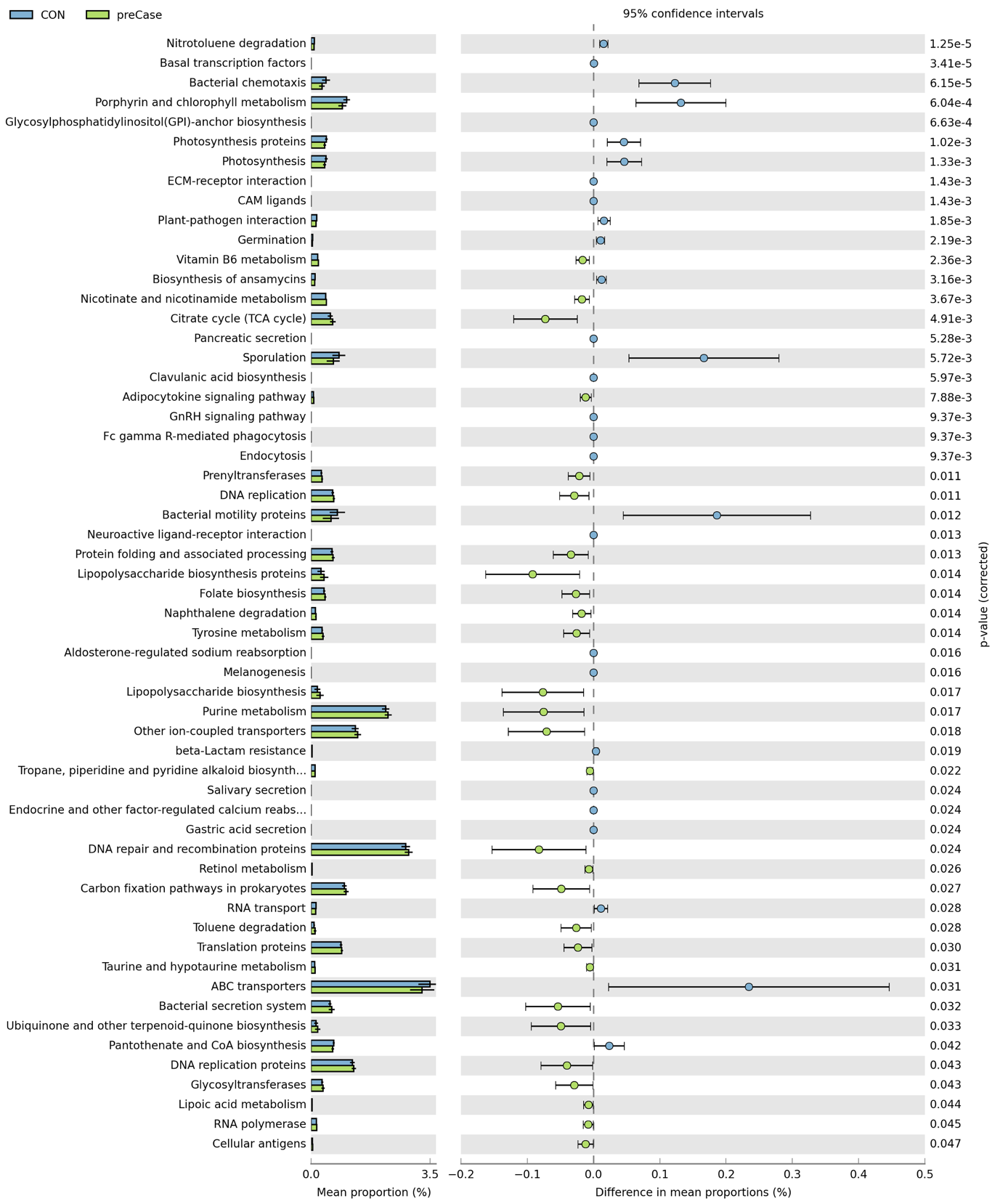
**
